# Editorial: ICF-Based Assessment and Documentation of Functioning and Disability

**DOI:** 10.3389/fresc.2022.877059

**Published:** 2022-04-26

**Authors:** Michaela Coenen, Soraya Maart, Thomas Maribo

**Affiliations:** ^1^Institute for Medical Information Processing, Biometry, and Epidemiology – IBE, Chair of Public Health and Health Services Research, LMU Munich, Munich, Germany; ^2^Pettenkofer School of Public Health, Munich, Germany; ^3^ICF Research Branch, Nottwil, Switzerland; ^4^Department of Health and Rehabilitation Sciences, Faculty of Health Sciences, University of Cape Town, Cape Town, South Africa; ^5^South African WHOFIC Collaborating Centre, MRC, Cape Town, South Africa; ^6^DEFACTUM, Central Denmark Region, Aarhus, Denmark; ^7^Department of Public Health, Aarhus University, Aarhus, Denmark

**Keywords:** functioning, disability, International Classification of Functioning Disability and Health, patient reported outcome, assessment

People with any kind of health condition such as acute or chronic disease or injury might experience impairments in body functions and structures, limitations in activities and restrictions in participation. Problems in functioning, that is disability, can change over time due to the natural course of the health condition, the effect of an intervention and features of the social and physical environment.

The World Health Organization's International Classification of Functioning, Disability and Health (ICF) (1) offers a framework and a classification to classify functioning and disability using its components body functions, body structures, activities and participation, as well as contextual factors. The ICF has been promoted as a classification system to generate comparable and standardized data. To ensure standardization it is necessary to develop and test assessments and documentation tools that can be implemented in clinical practice and research. Sound methodological approaches are also required to ensure the appropriate conceptualization, application and implementation of the ICF.

The aim of the Research Topic “*ICF-based Assessment and Documentation of Functioning and Disability*” is to comment on innovative uses of the ICF in documentation and assessment and to explore the application of these tools, including new methodologies to serve the needs of various research areas. A total of nine papers were accepted for this edition using review methodology (e.g., scoping review), mapping exercises, psychometric study designs, quantitative and qualitative study designs as well as mixed methods study designs.

The Research Topic contains four articles with a strong focus on methodology using the ICF as a framework. Cuenot's article maps the GEVA [Guide d'évaluation multidimensionnelle (2)] items used for the multidimensional assessment of the needs of persons with disabilities in France to the ICF, and identifies GEVA items not included in the ICF and which might be available for updating the ICF. Karhula et al. perform a scoping review aiming to shed light on personal factors defined in studies carried out in rehabilitation settings. Macdermid reports on the application of the ICF linking rules (3) and how these rules can be used to support content validation of PROMs. Newman-Griffis et al. investigate natural language processing (NLP) technologies to analyse patient functioning information recorded with claims for federal disability benefits in the United States into ICF domains.

In total, three articles included in this Research Topic report on the development and application of ICF-based tools in patients with acute and chronic diseases. Backmann et al. present their mixed methods study focusing on functioning in a sample of persons with self-reported disability following COVID-19 in Denmark. Björklund et al. report on how to use the ICF classification to describe how professionals in healthcare, habilitation and school document problems with everyday life functioning of children who completed treatment for a brain tumor. Scheel-Sailer et al. contributed to this Research Topic with a research article reporting on the development and implementation of an institutional standard of assessments relying on the ICF as a framework—the Nottwil Standard—for patients with newly acquired spinal cord injury.

The remaining two articles focus on psychometric properties of existing and newly developed ICF-based tools. Nielsen et al. report on the validity and clinical utility of the World Health Organization Disability Assessment Schedule 2.0 (WHODAS 2.0) (4) in older patients discharged from emergency departments. Stallinga et al. present their feasibility study on the usability of the preliminary ICF Core Set for patients after a hematopoietic stem cell transplantation from the perspective of nurses.

## Conclusion

There is sufficient evidence to support the use of the ICF as a framework in assessment and the further development of validated tools for patient reporting of limitations in functioning in various contexts.

## Author Contributions

All authors listed have made a substantial, direct, and intellectual contribution to the work and approved it for publication.

## Conflict of Interest

The authors declare that the research was conducted in the absence of any commercial or financial relationships that could be construed as a potential conflict of interest.

## Publisher's Note

All claims expressed in this article are solely those of the authors and do not necessarily represent those of their affiliated organizations, or those of the publisher, the editors and the reviewers. Any product that may be evaluated in this article, or claim that may be made by its manufacturer, is not guaranteed or endorsed by the publisher.
